# Respiratory and skin health among glass microfiber production workers: a cross-sectional study

**DOI:** 10.1186/1476-069X-8-36

**Published:** 2009-08-18

**Authors:** Penpatra Sripaiboonkij, Nintita Sripaiboonkij, Wantanee Phanprasit, Maritta S Jaakkola

**Affiliations:** 1 Institute of Occupational and Environmental Medicine, University of Birmingham, Edgbaston, Birmingham, UK; 2 Cancer Center, Ramathibodhi Hospital, Faculty of Medicine, Mahidol University, Bangkok, Thailand; 3 Department of Occupational Health and Safety, Faculty of Public Health, Mahidol University, Bangkok, Thailand; 4 Respiratory Medicine Unit, Dept. of Internal Medicine, Institute of Clinical Medicine, University of Oulu, P.O. Box 5000, FI-90014 University of Oulu, Oulu, Finland, and Oulu University Hospital, Oulu, Finland

## Abstract

**Background:**

Only a few studies have investigated non-malignant respiratory effects of glass microfibers and these have provided inconsistent results. Our objective was to assess the effects of exposure to glass microfibers on respiratory and skin symptoms, asthma and lung function.

**Methods:**

A cross-sectional study of 102 workers from a microfiber factory (response rate 100%) and 76 office workers (73%) from four factories in Thailand was conducted. They answered a questionnaire on respiratory health, occupational exposures, and lifestyle factors, and performed spirometry. Measurements of respirable dust were available from 2004 and 2005.

**Results:**

Workers exposed to glass microfibers experienced increased risk of cough (adjusted OR 2.04), wheezing (adjOR 2.20), breathlessness (adjOR 4.46), nasal (adjOR 2.13) and skin symptoms (adjOR 3.89) and ever asthma (adjOR 3.51), the risks of breathlessness (95%CI 1.68–11.86) and skin symptoms (1.70–8.90) remaining statistically significant after adjustment for confounders. There was an exposure-response relation between the risk of breathlessness and skin symptoms and increasing level of microfiber exposure. Workers exposed to sensitizing chemicals, including phenol-formaldehyde resin, experienced increased risk of cough (3.43, 1.20–9.87) and nasal symptoms (3.07, 1.05–9.00).

**Conclusion:**

This study provides evidence that exposure to glass microfibers increases the risk of respiratory and skin symptoms, and has an exposure-response relation with breathlessness and skin symptoms. Exposure to sensitizing chemicals increased the risk of cough and nasal symptoms. The results suggest that occupational exposure to glass microfibers is related to non-malignant adverse health effects, and that implementing exposure control measures in these industries could protect the health of employees.

## Background

Glass microfiber is a microfiber produced from glass at high temperature using a melting and fiberising process. It is one of the man-made vitreous fibers (MMVF), which are non-crystalline or vitreous in molecular structure [1] and widely used for thermal and acoustic insulation. The diameter of a microfiber is in the range of 0.2–4 μm [2]. Asbestos is a naturally occurring fiber that has been widely used for insulation and reinforcement of other materials. However, it is known to cause pleural diseases, lung fibrosis, lung cancer, mesothelioma, and chronic bronchitis in exposed workers [1,3]. Man-made synthetic fibers have been developed with the aim to reduce the asbestos-related health risks.

MMVF have been thought to cause fewer health problems than asbestos, but these have not been studied as extensively. Studies have suggested that glass fibers are possibly carcinogenic to humans [4,5], but recent reviews are cautious in making conclusions, because there is not enough evidence from human studies, so the conclusions are based mainly on evaluation of animal toxicology and mechanisms [1,6,7]. There are even fewer studies on potential non-carcinogenic respiratory effects of glass fibers [6,8,9] and these have provided inconsistent results. There is more consistent evidence of occurrence of skin diseases in glass fiber workers [10,11]. However, we were able to identify only one previous epidemiological study assessing the risk of skin symptoms related to occupational exposures in such industry [12].

**Figure 1:**
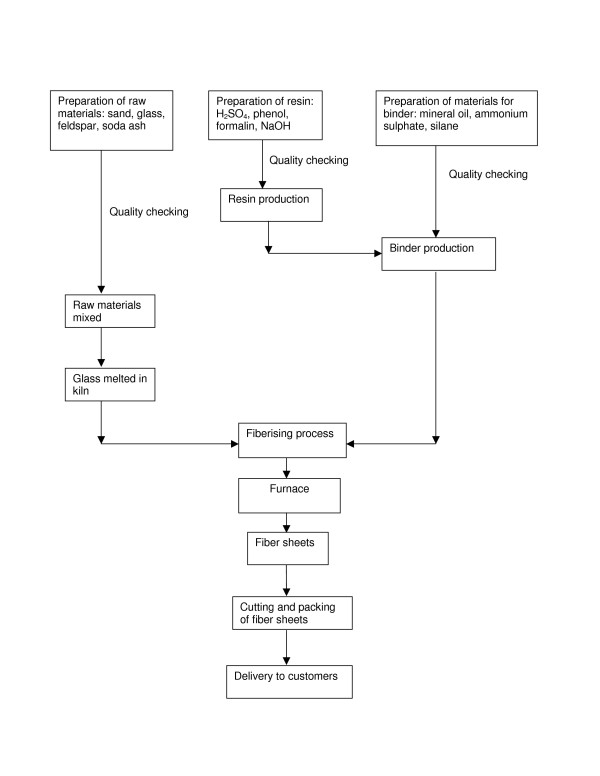
**Processes of glass microfiber production**.

Clinical practitioners and policy makers frequently face the question, whether there could be adverse health effects from exposure to glass microfibers. Because of the controversy concerning non-malignant health effects, we studied employees in a glass microfiber company in Thailand. Figure 1 shows the process of production. The process starts by preparing the raw materials, which include glass, feldspar, soda ash, and sand, for making molten glass, and by mixing chemicals, including sulfuric acid (H_2_SO_4_), phenol, sodium hydroxide (NaOH), and formalin, for making resin. A mixture of ammonium sulfate, mineral oil and silane is blended with resin to make a binder. The binder is transferred in a closed pump-operated tube to the fiberising machine. Glass is melted in a kiln and spun into microfibers, onto which the resin-binder is sprayed to strengthen the fibers. After that the coated microfibers are heated in a furnace in high temperature (1400–1600°C) to cure the resin and make sheets of fiber. In the final process the microfiber sheets are cut into the right size and wrapped in an aluminum foil. The quality of the microfiber sheets is checked before sending to customers. Exposure to microfibers of the workers occurs via two routes: by inhaling and by direct contact with the skin.

The aim of this study was to investigate the relations of occupational exposure to glass microfibers to respiratory and skin symptoms, asthma and spirometric lung function. Potential health effects related to exposure to sensitizers used in the process were also addressed.

## Methods

### Study design

A cross-sectional study was performed among employees of a glass microfiber production company and office workers from four companies in Thailand from September through December 2006. The study was approved by the ethics committee of the Faculty of Public Health at the Mahidol University, Thailand. Each participant gave an informed consent.

### Study population

All 102 workers of a microfiber factory (response rate 100%) participated in the study, including 14 workers preparing and mixing raw materials and chemicals, 15 furnace and fiberising process workers, 14 workers cutting fiber sheets, 35 packing workers, 10 warehouse workers, 5 quality control staff and 9 maintenance workers. Altogether 76 office workers (response rate 73%) from four factories formed the unexposed control population, including 18 from the microfiber factory, 24 from a milk powder factory, 18 from a wood furniture factory and 16 from a tile factory [13,14]. Office workers were managers, other administrative staff, chauffeurs, and security staff checking identification cards at the gates.

### Measurement methods

#### Questionnaire

A questionnaire on respiratory health was modified from the Finnish Environment and Asthma Study [15-20]. It inquired about personal characteristics, health information, smoking and exposure to secondhand smoke (SHS) at home and at work, occupation, exposures in the work environment, and exposures in the home environment. The questionnaire was translated into Thai and then back-translated into English to ensure the accuracy of the translation.

#### Lung function tests

All participants performed spirometry following the standards by the American Thoracic Society [21] with a Minato Autospiro PAL spirometer (Minato Medical Sciences Ltd, Osaka, Japan). The best forced expiratory volume in 1 second (FEV1) and forced vital capacity (FVC) out of a minimum of three acceptable forced expirations were used as outcomes. FEV1 and FVC as percentage (%) of predicted were calculated based on reference values from a healthy non-smoking Thai population [22].

#### Air measurements

The concentrations of respirable dust had been measured in the air of different factory areas by the gravimetric analysis method (NIOSH method 0600) in 2004 and 2005 by an independent consulting company [23]. Aluminum cyclones were used for 6 hours at the flow rate 2.5 l/min. Two environmental samples were taken in each area to get the average concentration. The concentrations of different chemicals, including NaOH, phenol, ammonia, formaldehyde and H_2_SO_4 _had also been measured by standard NIOSH methods [23]. There had not been any changes in the industrial processes or environmental control measures between 2004–5 and our data collection in 2006.

### Outcome assessment

The outcomes of interest included the occurrence of respiratory and skin symptoms in the past 12 months and the occurrence of asthma currently and ever. The symptoms included cough, phlegm production, wheezing, breathlessness, nasal symptoms, eye symptoms and skin symptoms, and they as well as asthma were defined based on answers to the questionnaire (Table 1).

**Table 1:** Definitions of symptoms and asthma used as outcomes

Symptoms/conditions	Definition
Cough	Recurrent or prolonged cough

Phlegm production	Recurrent or prolonged phlegm production

Wheezing	Wheezing or whistling of the chest

Breathlessness	Chest tightness or difficulty breathing

Nasal symptoms	Dryness, itching or smarting of nose, stuffy nose, runny nose or repeated sneezing (apart from colds)

Eye symptoms	Dryness of eyes, itchy eyes, irritation or smarting of eyes, watering of eyes, or redness of eyes

Skin symptoms	Dryness or flaking of skin, itchy skin, irritation, smarting or redness of skin, sore or tender skin, or urticaria

Current asthma	Asthma diagnosed by a physician in the past 12 months

Asthma ever	Asthma diagnosed by a physician ever during life time

Lung function outcomes included the best values of FEV1, FVC, FEV1 % of predicted and FVC % of predicted.

### Exposure assessment

Occupational exposure to glass microfibers was the main exposure of interest. This was assessed in two ways: 1) based on working as a factory worker (coded 1) or an office worker (coded 0, the reference category), and 2) factory workers were grouped into two exposure categories reflecting the level of microfiber exposure based on their job title and measurements of air respirable microfiber dust. High exposure group (n = 64) consisted of those working in the fiberising process, furnace, cutting and packing. Low exposure group (n = 38) consisted of those working in the raw material preparation, warehouse, maintenance, and quality control. These were compared to office workers forming the unexposed reference category.

Some factory workers had exposure to potentially sensitizing chemicals, such as formaldehyde, phenol resin and mineral oils (Figure 1), so we also analyzed separately potential effects related to exposure to any sensitizing chemical. The population was categorized into those having any exposure to sensitizers (n = 19) (coded 1), including material preparation and quality control staff, and into those with no exposure to sensitizers (n = 159) (coded 0), including the other factory as well as office workers.

### Data analysis

Odds ratios (OR) and 95% confidence intervals (95% CI) were calculated to quantify the relations between the exposures of interest and the occurrence of symptoms and asthma. All analyses were performed by SPSS statistical program, version 14. A model was fitted for each symptom outcome and for each type of exposure separately. All models adjusted for sex, age, educational level, smoking status (currently, formerly, never), and exposure to SHS at home and/or at work in multivariate logistic regression.

Linear regression analyses were used to estimate the relations between exposures of interest and lung function outcomes. A separate model was fitted for each lung function outcome and for each type of exposure separately. Age, sex, height, educational level, and smoking were adjusted for as potential confounders when studying FEV1 and FVC levels as outcomes, while FEV1 % predicted and FVC % predicted were already controlled for sex, age and height by the prediction equations.

## Results

### Characteristics and symptoms of the study population

Additional file 1 shows the characteristics of the study population. The population consisted of 123 males (69%) and 55 females (31%). The occurrence of upper and lower respiratory and skin symptoms and asthma are presented in table 2. Factory workers experienced about twice as much cough, breathlessness, nasal symptoms, and skin symptoms as office workers. The prevalence of wheezing and asthma were slightly higher in factory workers.

**Table 2:** Occurrence of respiratory and skin symptoms and asthma in factory and office workers

	Office workers N = 76		Factory workers N = 102	
**Symptom/condition**	**n**	**%**	**n**	**%**

Prolonged cough	13	17.1	37	36.3

Phlegm production	24	31.6	29	28.4

Wheezing	9	11.8	15	14.7

Breathlessness	12	15.8	42	41.2

Nasal symptoms	21	27.6	44	43.1

Eye symptoms	23	30.3	27	26.5

Skin symptoms	23	30.3	61	59.8

Asthma ever	3	3.9	6	5.9

Current asthma	0	0	5	4.9

### Air measurements

Air concentrations of respirable dust had been monitored in the factory in 2004 and 2005 (Table 3), the major component of this being glass microfibers. The concentrations were 0.07–1.70 mg/m^3^. The fiberising and furnace areas showed the highest mean concentration (1.70 mg/m^3 ^in 2005), while corridors (0.07 mg/m^3^) and warehouse area (0.31 mg/m^3^) had the lowest mean concentrations. Concentrations of other chemicals (mentioned in the methods) were in general low, with the exception that high concentration of ammonia had been measured in the fiberising process (3.48 mg/m^3^) and cutting (4.07 mg/m^3^) areas.

**Table 3:** Mean concentration of respirable dust (mg/m^3^) in different work areas

Area	2004	2005
Material preparation area	0.86	0.35

Fiberising and furnace area	-	1.70

Cutting area	1.52	0.50

Packing area	1.26	-

Warehouse area	0.31	-

Corridors	-	0.07

- means no measurements available

### Effects of glass microfibers and sensitizing chemicals on symptoms and asthma

The crude OR of cough (2.85, 95% CI 1.38–5.86), breathlessness (3.80, 1.83–7.92), nasal symptoms (2.06, 95% CI 1.08–3.91), and skin symptoms (3.45, 95% CI 1.83–6.49) were significantly increased in factory workers compared to office workers, while the OR of phlegm production and eye symptoms did not show any relation to factory work (Table 4). After adjusting for confounders, the ORs of breathlessness and skin symptoms remained significant (4.46, 1.68–11.86 and 3.89, 1.70–8.90, respectively). Adjusted OR of cough (2.04), wheezing (2.20), nasal symptoms (2.13) and ever asthma (OR 3.51) were also increased, but did not reach statistical significance. There were no subjects with current asthma among office workers, so the risk for this outcome could not be estimated.

**Table 4:** Odd ratios (OR) of respiratory and skin symptoms and asthma in relation to exposure to glass microfibers in factory workers compared to office workers

	All factory workers* N = 102		All factory workers* N = 102	
**Symptom/Disease**	**Crude OR**	**95% CI**	**Adjusted OR**^†^	**95% CI**

Cough	2.85	1.38–5.86	2.04	0.83–4.97

Phlegm	0.84	0.44–1.61	0.80	0.35–1.82

Wheezing	1.26	0.52–3.07	2.20	0.61–7.90

Breathlessness	3.80	1.83–7.92	4.46	1.68–11.86

Nasal	2.06	1.08–3.91	2.13	0.89–5.05

Eye	0.85	0.44–1.65	0.92	0.41–2.11

Skin	3.45	1.83–6.49	3.89	1.70–8.90

Asthma ever	1.52	0.37–6.29	3.51	0.29–55.99

*Office workers formed the reference category (OR = 1)

^†^Adjusted for sex, age, education, smoking, and exposure to SHS at work and/or at home

Table 5 presents adjusted ORs according to the level of exposure to glass microfibers. Both low and high exposures were related to significantly increased risk of breathlessness (adjusted OR 3.94, 95% CI 1.33–11.71, and 5.08, 1.69–15.23, respectively) and skin symptoms (2.99, 1.13–7.91, and 4.82, 1.89–12.33, respectively), the trend suggesting exposure-response relation. The ORs of wheezing, nasal symptoms and asthma ever were increased, although not statistically significant. The risk of these symptoms did not show any obvious exposure-response relation.

**Table 5:** Adjusted odd ratios (OR) of respiratory and skin symptoms and asthma in relation to the level of microfiber exposure and exposure to sensitizing chemicals

	Low MF level* N = 38		High MF level* N = 64		**Exposure to any sensitizer**^†^ N = 19	
**Symptom/Disease**	**Adjusted OR**^‡^	**95% CI**	**Adjusted OR**^‡^	**95% CI**	**Adjusted OR**^‡^	**95% CI**

Cough	1.72	0.61–4.88	2.34	0.87–6.31	3.43	1.20–9.87

Phlegm	0.55	0.20–1.52	1.08	0.42–2.76	0.61	0.19–1.92

Wheezing	2.38	0.58–9.79	2.00	0.46–8.70	1.28	0.30–5.50

Breathlessness	3.94	1.33–11.71	5.08	1.69–15.23	2.84	0.99–8.12

Nasal	2.01	0.74–5.44	2.25	0.84–6.00	3.07	1.05–9.00

Eye	0.73	0.26–2.02	1.11	0.44–2.83	0.91	0.30–2.78

Skin	2.99	1.13–7.91	4.82	1.89–12.33	1.95	0.65–5.80

Asthma ever	3.48	0.18–66.84	3.52	0.21–58.07	1.41	0.12–16.38

*Office workers formed the reference category (OR = 1)

^†^Compared to office workers and factory workers with no exposure to sensitizers (OR = 1 in this reference category)

^‡^Adjusted for sex, age, education, smoking, and exposure to SHS at work and/or at home

MF = glass microfiber exposure

Table 5 also shows adjusted ORs in relation to exposure to any sensitizers. Such exposure was related to significantly increased risk of cough (3.43, 95% CI 1.20–9.87) and nasal symptoms (3.07, 1.05–9.00). The risk of breathlessness was also increased (2.84, 0.99–8.12), although it did not reach statistical significance.

### Effects of glass microfibers and sensitizers on lung function

No significant deficits in lung function were detected in relation to glass microfiber or sensitizer exposure, although small deficits were seen in FEV1 and FEV1 % predicted in the high microfiber exposure group (Table 6).

**Table 6:** Adjusted effects on lung function related to exposure to glass microfibers in all factory workers, by the level of microfiber exposure and exposure to sensitizing chemicals

Lung function parameter	**All factory workers**^‡^ N = 102		**Low MF level**^‡^ N = 38		**High MF level**^‡^ N = 64		**Exposure to any sensitizer**^§^ N = 19	
	**β**	**95% CI**	**β**	**95% CI**	**β**	**95% CI**	**β**	**95% CI**

FEV1* (liter)	0.05	-0.10 to 0.20	0.13	-0.04 to 0.31	-0.01	-0.18 to 0.15	0.24	0.04 to 0.43

FVC* (liter)	0.14	-0.02 to 0.30	0.19	-0.007 to 0.38	0.10	-0.07 to 0.29	0.29	0.08 to 0.50

FEV1%pred^†^	1.20	-3.35 to 5.75	2.68	-2.74 to 8.11	-0.05	-5.24 to 5.14	4.64	-1.53 to 10.80

FVC%pred^†^	1.81	-2.33 to 5.94	1.85	-3.09 to 6.79	1.77	-2.95 to 6.49	4.37	-1.23 to 9.98

* Effect estimates adjusted for sex, age, height, education, and smoking

^†^Effect estimates adjusted for education and smoking. The predicted values controlled for age and height. They were calculated separately for men and women

^‡^Compared to office workers (OR = 1 in this reference category).

^§^Compared to office workers and factory workers with no exposure to sensitizers (OR = 1 in this reference category)

MF = glass microfiber exposure

## Discussion

Glass microfiber is one of the synthetic man-made vitreous fibers that are widely used for insulation. Because of the controversy concerning potential non-malignant health effects of glass microfibers, we studied workers in a factory producing glass microfiber sheets in Thailand. Factory workers experienced increased risk of cough, wheezing, breathlessness, nasal symptoms, skin symptoms and ever asthma compared to office workers. After adjusting for potential confounders, the risk of breathlessness and skin symptoms remained significantly increased, and the risk of both of these symptoms showed a trend suggesting exposure-response relation with increasing glass microfiber exposure level. Interestingly, these effects were detected at concentrations of respirable dust, consisting mainly of glass microfibers, that were below the threshold limit value for respirable dust of 3 mg/m^3^.

The respiratory and skin effects observed could be due to an irritant mechanism by glass microfibers. Lack of effect on spirometric lung function would be consistent with an irritant effect. A review by the World Health Organization (WHO) suggested that MMVF can cause transient irritation of skin, eyes and upper airways, but that there has been so far insufficient evidence of effects on lower airways [24]. In our study, the risks of wheezing (adjusted OR 2.20) and asthma (adjusted OR 3.51) were also increased, although not statistically significantly, which raises the question whether exposure to glass microfibers could induce an irritant-type of asthma [25]. Our study is not able to answer this question definitely, because of the small number of asthmatics in our study population. Abbate and co-workers [26] investigated 29 men employed in glass fiber reinforced plastic processing with a clinical check-up and bronchoalveolar lavage (BAL). Microscopic and biochemical analyses of BAL fluid suggested that oxidative stress activates an inflammatory process in the airways as a consequence of exposure to glass fibers.

Some processes of the glass microfiber production involve working with potential sensitizers, such as formaldehyde. Exposure to sensitizing chemicals was associated with significantly increased risks of cough and nasal symptoms. The risk of breathlessness was also increased, but no obvious effect on skin symptoms was detected. Exposure to sensitizing chemicals did not affect adversely spirometric lung function or the risk of asthma, but these conclusions have to be cautious because of the small number of workers with sensitizer exposure (n = 19).

### Validity issues

The small sample size of office workers is a limitation of this study. The number of subjects that could be studied was dependent on access to workforces, a problem commonly encountered in occupational epidemiology. To counterbalance this limitation, we made efforts to get good response rates and succeeded in this well. The response rate was 100% among glass microfiber factory workers currently employed at the factory and it was also relatively good at 73% among office workers, so selection bias is not likely to explain our results.

The outcome assessment in this study was based on self-report of symptoms and doctor-diagnosed asthma and measurements of lung function. All of these investigations were carried out in the same way in factory and office workers. The same protocol for the questionnaire and spirometry was applied in all four factories, where office workers were recruited to form the control group. It is possible that our study underestimates to some extent the true effects of glass microfiber exposure because of the cross-sectional study design. It has previously been estimated that about 5% of workers involved in MMVF production leave employment because of problems of skin irritation [2], meaning that those staying in the industry are likely to be selected based on better health. Our finding that vital capacity was actually somewhat better in factory workers than in office workers supports the possibility of some 'healthy worker' selection taking place. A small number of asthmatics, especially current asthmatics among office workers, is a limitation of the study, as the results suggested increased risk of ever asthma in relation to glass microfiber exposure, but this did not reach statistical significance. The rather small number detected also among factory workers, although the risk of wheezing was increased, may reflect the health care system. Workers may not seek medical help for their asthma-type symptoms because of fear of losing their job.

Exposure assessment was based on job title complemented by air respirable dust measurements from 2004 and 2005. There had not been any changes in the industrial processes or environmental control measures between these years and our data collection in 2006, so the air measurements are likely to reflect well the relevant exposure situation in the factory. These methods give a rough estimate of microfiber exposure level, which enabled us to compare high and low exposure groups. The unexposed control group (i.e. office workers) was formed of those working for the companies, but not working in the production areas, packing areas, warehouse or quality control, i.e. not having any major exposures. However, the managers sometimes visited the process for short periods of time. During these visits they were required to wear protective masks. It is possible that some of them could have had small amount of exposure, which could cause some underestimation of the true effects of glass microfiber exposure.

The number of workers exposed to sensitizing chemicals was small (n = 19), but such exposure showed significant associations with cough and nasal symptoms. Sensitizer exposure was related to a somewhat different symptom pattern than glass microfiber exposure, but most of those exposed to sensitizers were also exposed to microfibers, so we were not able to disentangle their effects sharply.

We collected data on many potential confounders in our questionnaire, including personal characteristics (sex, age), genetic background (parental atopy or asthma), socio-economic status (education), lifestyle habits (smoking), and other risk factors at work (SHS exposure, stress at work) and at home (pet keeping). We addressed all of these and controlled for the factors that were important potential confounders in the multivariate regression models in order to exclude them as potential explanations for our findings.

### Synthesis with previous knowledge

Only a few previous studies have been reported on non-cancer respiratory effects of glass fibers and they have provided inconsistent results. According to reviews there has been insufficient evidence to make any firm conclusions [6], so more studies on this topic in human subjects are needed.

In the only previous epidemiological study on respiratory effects, Moulin and co-workers [9] investigated 2,024 men from three plants manufacturing glass wool and two manufacturing rock wool in France. In the biggest plant studied, exposure to fibers was related to significantly increased risk of cough, phlegm, dyspnea and pharynx-larynx symptoms, ORs being 1.6–6.0. However, no effects were detected in the other four factories. Working in resin preparation was consistently related to the risk of pharynx-larynx symptoms. Thus, the findings in the largest factory are consistent with our findings.

Results of three clinical studies on non-malignant respiratory effects in workers exposed to glass fibers are partly compatible with our results. A study of workers of a filament glass fiber plant in UK identified 7 cases who had work-related asthma in serial PEF measurements, but was not able to identify the causal agent in challenge testing [27]. An investigation of a fiberglass wool insulation production facility in USA was prompted because of high prevalence of wheezing and use of asthma medication among employees, but these problems seemed to be related to endotoxin from bacteria growing in the recirculating wash water [28]. Kilburn and co-workers [29] studied clinically 284 workers from Sheet metal Workers' International Association who had at least 20 years of exposure to fiberglass. Nineteen percent of the study population reported throat irritation, 13% nose irritation and 10% chest burning. Spirometric lung function was reduced in fiberglass-exposed workers compared to the reference values. However, some of these study subjects had also had exposure to asbestos and there was no control population for comparison to assess the risks of these conditions.

Some studies have investigated mortality from non-malignant respiratory disease in workers exposed to MMVF. Chiazze and coworkers [30] conducted a case-control study among workers employed at the Owens-Corming Fiberglass plant in Ohio and reported a non-significantly increased adjusted OR 1.50 (95% CI 0.55–4.08) in relation to cumulative exposure to >300 fibers/ml. A longitudinal study of MMVF workers from 13 factories in seven countries followed 11,373 workers and found a slightly increased mortality from bronchitis, emphysema and asthma among glass wool workers with a SMR 1.12 (95% CI 0.82–1.49) [31].

Workers are also exposed to glass microfibers through direct contact with the skin. Skin symptoms, such as itching, and dermatitis are rather consistently reported in relation to occupational exposure to glass microfibers or other MMVFs in case reports, studies of individual factories, and registry-based studies [10-12,32]. Glass fibers and sensitizers, such as phenol-formaldehyde resin, have been identified as causes for individual cases. According to the Finnish Register of Occupational Diseases, the rate of skin diseases due to MMVF was highest in insulation workers with an annual incidence of 9.1 per 100,000 employed workers [11]. Only one previous study assessed the risk of skin diseases related to glass microfiber exposure. A Japanese cross-sectional study of 148 workers from fiberglass reinforced plastics factories [12] found significantly increased risk of work-related skin problems with OR ranging between 4.10 and 4.69. Thus, the Japanese study is consistent with our study that shows an OR of 3.89 for skin symptoms in factory workers compared to office workers.

## Conclusion

This study provides evidence that exposure to glass microfibers increases the risk of both respiratory and skin symptoms, increasing exposure showing exposure-response relation with breathlessness and skin symptoms. The risks of wheezing and asthma were also increased in relation to glass microfiber exposure, although not statistically significant, probably because of a small number of asthmatics in our study population. Exposure routes via inhalation and direct contact with skin could both be of importance. Glass microfibers are considered irritant, but some sensitizing chemicals, such as formaldehyde, are also used in the manufacturing processes. Exposure to these sensitizers increased the risk of cough and nasal symptoms. The results suggest that occupational exposure to glass microfibers is related to non-malignant adverse health effects, and that implementing exposure control measures in this type of industries could protect the health of employees.

## Abbreviations

The abbreviations used in the text and tables are the following: BAL: Bronchoalveolar lavage; CI: Confidence interval; FEV1: Forced expiratory volume in one second; FVC: Forced vital capacity; H_2_SO_4_: Sulfuric acid; MF: Glass microfiber; MMVF: Man-made vitreous fiber; mg/m^3^: Milligram per cubic meter; NaOH: Sodium hydroxide; NIOSH: National Institute of Occupational Safety and Health; OR: Odds ratio; SHS: Second-hand tobacco smoke.

## Competing interests

The authors declare that they have no competing interests.

## Authors' contributions

PS designed the study and data collection together with MSJ, conducted the data collection, analyzed the data, and drafted the manuscript. NS helped with the data analysis and revision of the manuscript. WP helped with the data collection and revision of the manuscript. MSJ designed the study and data collection with PS, planned the data analysis, and helped with interpretation of the data and drafting the manuscript. All authors have approved the final version of the manuscript.

## Supplementary Material

Additional file 1 **Characteristics of the study population**. Characteristics of the study population. Click here for file
