# Supplementary material for: Respiratory and skin health among glass microfiber production workers: a cross-sectional study
Source: Environ Health. 2009 Aug 18;8:36. doi: 10.1186/1476-069X-8-36 (PMC2739167; doi:10.1186/1476-069X-8-36)
Supplement: Additional file 1 — Characteristics of the study population. Characteristics of the study population. [file 1476-069X-8-36-S1.doc]

**ADDITIONAL FILE 1.** Characteristics of the study population

|  | **Office workers**  **N = 76** | | **Factory workers**  **N = 102** | | **Total**  **N = 178** | |
| --- | --- | --- | --- | --- | --- | --- |
|  | **n** | **%** | **n** | **%** | **n** | **%** |
| Sex |  |  |  |  |  |  |
| Male | 35 | 46.1 | 88 | 86.3 | 123 | 69.1 |
| Female | 41 | 53.9 | 14 | 13.7 | 55 | 30.9 |
| Age |  |  |  |  |  |  |
| < 25 years | 9 | 11.8 | 6 | 5.9 | 15 | 8.4 |
| 26 – 45 years | 52 | 68.4 | 61 | 59.8 | 113 | 63.5 |
| > 46 years | 15 | 19.8 | 35 | 34.3 | 50 | 28.1 |
| Education |  |  |  |  |  |  |
| Primary or high school | 29 | 38.1 | 74 | 72.6 | 103 | 57.9 |
| Vocational training or  college | 17 | 22.4 | 23 | 22.5 | 40 | 22.5 |
| Bachelor or higher  university degree | 30 | 39.5 | 5 | 4.9 | 35 | 19.6 |
| Smoking status* |  |  |  |  |  |  |
| Never | 55 | 75.4 | 46 | 45.1 | 101 | 57.7 |
| Past | 5 | 6.8 | 20 | 19.6 | 25 | 35.3 |
| Current | 13 | 17.8 | 36 | 35.3 | 49 | 28.0 |
| Secondhand smoke exposure at work and/or at home | 32 | 42.1 | 57 | 55.9 | 89 | 50.0 |

^*^Information on smoking status was missing for 3 subjects
